# Surveillance of Antimicrobial Resistance in the Asian Seabass (*Lates calcarifer*) Supply Chain Using Nanopore Sequencing

**DOI:** 10.3390/foods14101691

**Published:** 2025-05-10

**Authors:** Matsapume Detcharoen, Panatda Khrueakaew, Soottawat Benjakul, Chonticha Romyasamit, Watcharapol Suyapoh, Jirakrit Saetang

**Affiliations:** 1 Division of Biological Science, Faculty of Science, Prince of Songkla University, Hat Yai, Songkhla 90110, Thailand; matsapume.d@psu.ac.th; 2 International Center of Excellence in Seafood Science and Innovation, Faculty of Agro-Industry, Prince of Songkla University, Hat Yai, Songkhla 90110, Thailand; panatdakhruaekeaw11@gmail.com (P.K.); soottawat.b@psu.ac.th (S.B.); 3 School of Allied Health Sciences, Walailak University, Nakhon Si Thammarat 80160, Thailand; chonticha.ro@wu.ac.th; 4 Veterinary Pathology Unit, Department of Veterinary Science, Faculty of Veterinary Science, Prince of Songkla University, Hat Yai, Songkhla 90110, Thailand; watcharapol.s@psu.ac.th

**Keywords:** antibiotics, barramundi, metagenomics, one health, resistome

## Abstract

Intensive fish farming worldwide has increased reliance on antibiotics to control bacterial pathogens, raising concerns about antimicrobial resistance (AMR) in aquaculture. These resistant bacteria can persist and pass through the food supply chain, from farms to consumers. Despite this risk, antimicrobial resistance genes (ARGs) in aquaculture environments and fish products have not been elucidated. This study aimed to detect ARGs found in the Asian seabass (*Lates calcarifer*), an economically important fish in Thailand, collected from farms, fish container vehicles, and markets, using Nanopore metagenomic sequencing. We detected multiple ARGs in all sample types. Water samples harbored the *rpsL* gene conferring streptomycin resistance. Container samples exhibited the highest diversity of ARGs, including multiple beta-lactamases and the *rsmA* gene, conferring resistance to fluoroquinolones, diaminopyrimidines, and phenicol antibiotics. Fish samples generally lacked ARGs, except for one sample harboring *rsmA*. Non-metric multidimensional scaling revealed distinct microbial communities in water, compared with those found in container and fish samples, indicating potential cross-contamination during handling or storage. Our findings emphasize that containers could be critical control points for minimizing AMR spread. Overall, this study highlights the interconnection between environmental, fish, and human health, highlighting the importance of integrated AMR surveillance and management in aquaculture systems.

## 1. Introduction

Antimicrobial resistance (AMR) has emerged as a critical global health threat, which the food chain plays a major role in its development and transmission. AMR occurs when bacteria develop genetic mutations or acquire resistance genes through horizontal gene transfer that allow them to survive when exposed to antibiotics [1]. The food chain plays a significant role in the spread of AMR through various interconnected pathways. Resistant bacteria can be transferred into humans via consumption of contaminated food products, direct contact with food-producing animals, or indirectly through environmental exposure to animal waste and agricultural runoff [2]. The extensive use of antibiotics in food animal production, both for disease treatment and growth promotion, creates selective resistance of bacteria toward antibiotics. Antimicrobial usage in livestock has been shown to increase the prevalence of resistant bacteria not only in animals but also in human populations through the food supply chain [3,4]. The antibiotic-resistant bacteria from food animals can colonize the human gut through the food chain, potentially sharing resistance genes with human pathogens [5]. The complex and multifaceted nature of AMR spread through the food system necessitates the integrated “One Health” approaches by the disruption of interconnections between human, animal, and environmental health.

Fish aquaculture has a profound economic role in Southeast Asia. Intensive farming and overcrowding have led to bacterial infections among fish stocks. Antibiotics such as ampicillin, chloramphenicol, and quinolones are frequently used to treat bacterial infections [6]. Unfortunately, misuse and overuse of these drugs due to insufficient knowledge have accelerated the emergence of antibiotic-resistant bacteria and antibiotic resistance genes (ARGs) in aquaculture environments [7]. These resistant microbes pose serious threats to human health as they can persist through the food supply chain, from farms to consumers. Aquaculture now accounts for nearly half of global seafood production, and Asian countries contribute about 90% [8]. The rapid intensification of farming practices has been accompanied by increasing antimicrobial use. Some fish species, such as catfish, have higher usage rates per kilogram than terrestrial animals [8]. Moreover, concern about resistance to medically important antimicrobials in foodborne pathogens isolated from aquaculture environments, particularly in Asia, has been missing [8]. Many countries do not have strict regulatory frameworks to control antibiotic use in aquaculture, leading to awareness of antibiotic residues in products and ecological impacts [9,10]. Of the top five aquaculture-producing countries globally, only three nations currently have national action plans that specifically address antimicrobial resistance in aquaculture [11]. Thus, there is an urgent need for a unified global effort to regulate antibiotic use and monitor resistance risks in aquaculture.

Asian seabass, also known as barramundi (*Lates calcarifer*), is commercially farmed in Southeast Asia and Australia [12]. The Asian sea bass market is projected to grow at a compound annual growth rate (CAGR) of 4.5% between 2024 and 2034, reaching an estimated value of USD 1,567 million by the end of the forecast period [13]. The fish is highly fecund, fast-growing, and can inhabit freshwater, brackish, and marine environments [14]. Since the species is commonly cultivated in floating cages and concrete tanks [15], disease management remains a significant challenge in commercial production. Bacterial infections pose a particular threat to aquaculture fish. Several diseases linked to Vibrionaceae have been identified as among the most prevalent illnesses significantly threatening Asian seabass productivity [16]. Simultaneously, the development of antimicrobial resistance in over 98.5% of bacterial isolates from Asian seabass farms has been documented, with multidrug resistance patterns [17]. However, the information regarding the presence and spread of AMR throughout the Asian seabass supply chain is still lacking, thus leaving a critical knowledge gap.

There is growing concern that resistant microbes can persist throughout the food chain, from farms to consumers, thereby impacting human health. This study aimed to determine ARGs in Asian seabass throughout the supply chain, including farms, fish containers, and markets, using Oxford Nanopore metagenomic sequencing. This study not only provided AMR in Asian seabass but also served as a model for a One Health approach to AMR surveillance in aquaculture.

## 2. Materials and Methods

### 2.1. Sample Collection and DNA Extraction

Water samples from both outside and inside the fishing cages were collected using a vertical water sampler at a depth of one meter. For each location, three replicates of one liter each were obtained. Each sampling spot was at least 10 meters apart. The samples were stored on ice and transported to the laboratory on the same day. Each sample was filtered through a 0.2 μm filter in a clean room [18,19]. DNA was extracted from the filter using the DNeasy PowerWater Kit (Qiagen, Hilden, Germany) according to the manufacturer’s instructions. A blank sample was also prepared during the extraction process and used as a negative control. The quantity and quality of the extracted DNA were assessed using a Nanodrop spectrophotometer and agarose gel electrophoresis. All the DNA samples were stored at –20 °C.

The samples from the transport vehicles and storage containers were collected using a 3M Sponge-Stick SSL10DE sampling sponge pre-hydrated with 10 mL of neutralizing broth (3M, Saint Paul, MN, USA) by swabbing the internal walls of each truck (sides, door, and floor) or containers 20 times on the front and back of each sponge. Sponges offer a practical means to sample relatively large surface areas and increase the chance of detecting distributed contaminants [20,21]. Three samples were collected from three different containers at each site. The sponges were stored in the supplied neutralization buffer and kept on ice. Each sponge was agitated in a stomacher for 1 min at 250 rpm. The resulting liquid was used for DNA extraction using the same method as the water samples. Another clean sponge was used for the negative control.

Fresh dead fish with no signs of decay were purchased directly from local markets, one fish per market, to evaluate the presence of antibiotic residues at the point of sale and during transportation. Vendors were asked to confirm that these fish did not originate from the farms where our earlier samples had been obtained. Samples were stored on ice until reaching the laboratory and processed similarly to Rheman et al., 2024 [22]. First, five gill-slit tissue sections were excised from each side, along with 10 mg of flesh, including skin, from both sides. DNA extraction was done using the DNeasy Blood & Tissue Kit (Qiagen, Hilden, Germany) according to the manufacturer’s instructions. Gill slits, continuously bathed in water, are recognized reservoirs for water-borne bacteria [23,24]. Second, to target exogenous microbes that may be acquired during handling and distribution [25], the external surface was sampled by swabbing left and right flanks, starting from the anterior end and moving posteriorly along the lateral line. This swabbing process was repeated ten times on each side to ensure thorough mucus collection. The swabbed cottons were put in a stomacher bag and homogenized for 1 min at 250 rpm, followed by the DNA extraction using the same kit. DNA extracted from different parts of the same fish was then pooled for sequencing.

### 2.2. Library Preparation and Sequencing

Approximately 400 ng of DNA from each sample was end-prepped using the NEBNext Companion Module for Oxford Nanopore Technologies (NEB, Ipswich, MA, USA) and purified with AMPure XP Beads (Beckman Coulter, Indianapolis, IN, USA). A DNA barcode was attached to each sample using the Native Barcoding Kit 24 v14 (SQK-NBD114.24, Oxford Nanopore Technologies, Oxford, UK) following the manufacturer’s protocol. The barcoded samples were then pooled, and sequencing adaptors were attached. The library was sequenced using a Nanopore MinION device with a 10.4.1 flow cell for 48 h. One flow cell was used to sequence all water samples, another flow cell for all container samples, and two fish samples were sequenced on separate flow cells.

### 2.3. Bioinformatic Analysis

Raw sequence data were basecalled and demultiplexed using Dorado v0.7.2 (Oxford Nanopore Technologies, Oxford, UK) with the high accuracy model v5.0.0. Reads with less than 150 bases were discarded using Fastp v0.23.4 [26]. To access microbial diversity in each sample, Kraken2 v2.1.3 [27] was used to classify the reads against the standard_prebuilt_pluspf_2024-01-12 database with a confidence value of 0.2 on Galaxy [28]. The taxonomy and abundances were imported into the Phyloseq v1.48.0 package [29] in R v4.4.1 [30]. For alpha diversity, the Shannon diversity index of each sample was calculated. Significant differences between sample groups (water, containers, and fish) were tested using the Kruskal–Wallis test and pairwise Wilcoxon rank sum test. For beta diversity, non-metric multidimensional scaling (NMDS) based on Bray–Curtis dissimilarity distance was employed. Permutational multivariate analysis of variance (PERMANOVA) in vegan v2.6-6.1 [31] was used to test for significant differences among groups. An alpha of 0.05 was used throughout the analysis. The taxonomic profiles generated with Kraken2 were carefully checked for common bacterial pathogens reported by the European Food Safety Authority and the European Centre for Disease Prevention and Control [32,33], such as *Salmonella* spp., *Campylobacter* spp., *Escherichia coli*, *Staphylococcus aureus*, *Yersinia* spp., *Listeria* spp., and *Brucella* spp., to determine whether these bacteria were present in the samples. For AGRs detection, the filtered reads of each sample were assembled using Flye v2.9.5-b1801 [34] with the metagenome option. The assembled contigs were used to predict ARGs using the Resistance Gene Identifier (RGI-main) v6.0.3 against the Comprehensive Antibiotic Resistance Database (CARD) v3.3.0 [35] with low quality, perfect, and strict options. Only genes with more than or equal to 80% identity were included for analysis.

## 3. Results

For each farm, water from outside and inside the fishing cages and containers, especially insulated boxes, was collected (Table 1). Fish samples were collected from eight vendors in six local markets. DNA sequencing generated mean reads of 1,259,535 reads for water samples, 2,467,644 reads for container samples, and 8,062,848 reads for fish samples. Negative controls using two different DNA extraction kits produced 94,500 and 54,477 reads, respectively (Table S1).

### 3.1. Bacterial Diversity

**Figure 1:**
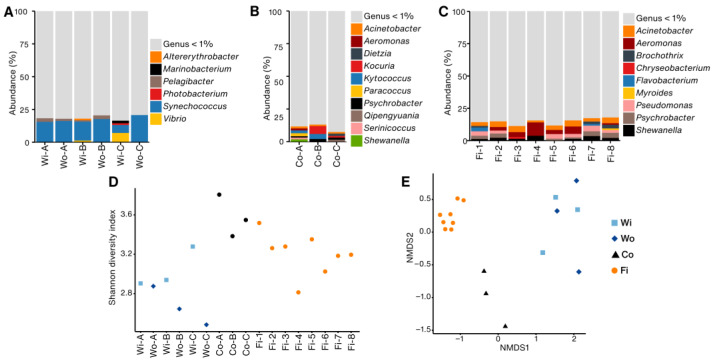
Relative abundance of bacterial genera, alpha and beta diversity across the samples. (**A**) Water collected inside (Wi) and outside (Wo) the fishing cages of farms A, B, and C. (**B**) Fish containers from farms A, B, and C. (**C**) Fish samples. Genera representing less than one percent of the sample were grouped together and labeled as “Genus < 1%”. (**D**) Shannon diversity index. (**E**) Non-metric multidimensional scaling (NMDS) based on Bray–Curtis dissimilarity matrix showing stress value of 0.06.

Taxonomic profiling showed that *Synechococcus* dominated the bacterial communities in most water samples, accounting for 6–20% of total reads (Figure 1A). Both inside (Wi) and outside (Wo) water samples from Farms A and B were similar, consistently containing low proportions of *Altererythrobacter*, *Marinobacterium*, *Pelagibacter*, and *Photobacterium*. However, water inside the cage of Farm C (Wi-C) showed a higher abundance of *Vibrio* (7%) than the outside water (Wo-C, <0.2%). Across all water samples, approximately 80% of bacterial genera were either unclassified or detected at very low relative abundances, each accounting for less than 1% of the total bacterial community composition.

Fish container samples displayed different bacterial communities compared with the water samples (Figure 1B). *Kocuria*, such as *Kocuria rosea* and *Kocuria turfanensis*, had the highest abundance in Co-B (6%). *Acinetobacter*, *Paracoccus*, and *Psychrobacter* were consistently present across containers at low abundance (<2%).

Around 100 bacterial genera were found across the fish samples. *Acinetobacter* (1–4%) and *Shewanella* (1–3%) were present in all fish samples (Figure 1C). Similarly, *Aeromonas* was also found in all samples, and the highest relative abundance (10%) was found in Fi-4, ranging from 0.7 to 5.7% in other samples. Other genera, including *Brochothrix*, *Chryseobacterium*, *Flavobacterium*, and *Pseudomonas*, were present at lower abundances, collectively representing about 1–8% of the bacterial community. At the species level, *Acinetobacter johnsonii*, *Aeromonas salmonicida*, *Vibrio harveyi*, and *Shewanella baltica*, were present in all fish samples (Table S2).

Shannon diversity index at the species level revealed that water, container, and fish samples differed significantly (Wilcoxon rank-sum test, *p* < 0.05). However, there was no difference between the water inside and outside the cages (Wilcoxon rank-sum test, *p* = 0.08). Beta diversity using NMDS analysis revealed two different clusters of bacterial communities (PERMANOVA, *p* = 0.004). The first cluster contained exclusively water samples, and the second cluster included containers and fish samples. The stress value of 0.06 strongly supported the distinction between groups (Figure 1E).

*Salmonella*, *Campylobacter*, *Listeria*, *Brucella*, and *S. aureus* were detected at very low abundance, with each being lower than 10 reads. In contrast, *E. coli* was predominantly found across most samples, exhibiting high prevalence in fish, particularly in samples Fi-3 and Fi-6. *Yersinia* was mainly identified in fish. The highest prevalence was observed in sample Fi-4.

### 3.2. Antimicrobial Resistance Genes

**Figure 2:**
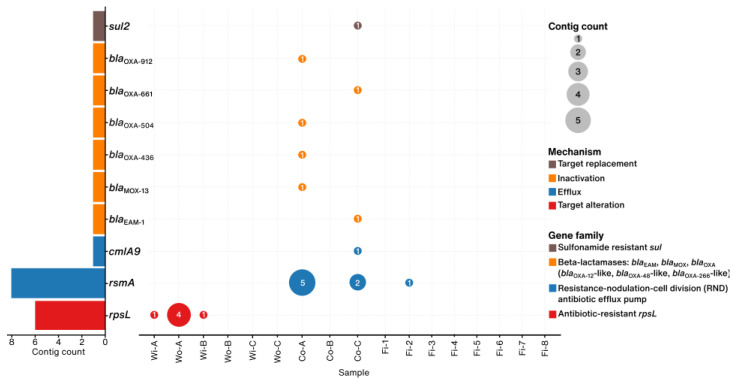
Distribution of antibiotic resistance genes (ARGs) across water, container, and fish samples, categorized by resistance mechanism and gene family. The size of the circles represents the number of contigs containing each ARG, and the color indicates the resistance mechanism and gene family.

ARGs were detected across all sample types (Figure 2). The *rpsL* gene, belonging to the antibiotic-resistant *rpsL* family, was found exclusively in water samples. Sample Wo-A exhibited the highest abundance of this gene, presented in four contigs, whereas samples Wi-A and Wi-B each contained a single instance. Container samples revealed a broader range of ARGs, including the *rsmA* gene, associated with the resistance-nodulation-cell division (RND) antibiotic efflux pump family. This gene was the most prevalent, particularly in sample Co-A. Additional ARGs detected in container samples included *beta-lactamase* genes, such as *bla*_OXA_ variants (*bla_OXA-436_* [*bla*_OXA-48_-like], *bla*_OXA-504_*, bla*_OXA-661_ [*bla*_OXA-266_-like], and *bla*_OXA-912_ [*bla*_OXA-12_-like]), as well as *MOX-13* and *EAM-1*. ARGs with low abundance, including *cmlA9* and *sul2*, were also found in these samples. In contrast, fish samples showed low ARG diversity, in which only sample Fi-2 contained *rsmA*.

### 3.3. Gene Family and Resistant Drug Class of ARGs

**Figure 3:**
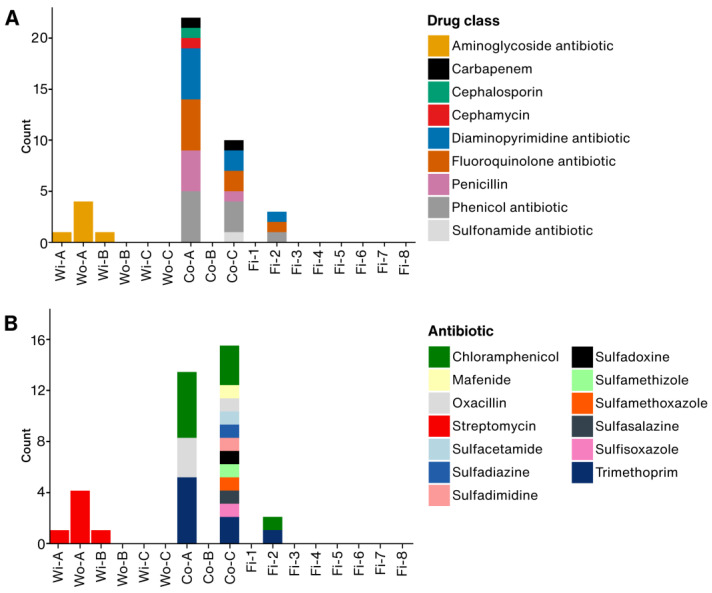
Distribution of antibiotic-resistance-genes (ARGs) annotations in the metagenomic contigs recovered from each sample based on (**A**) drug class and (**B**) specific antibiotics. For every contig that matched an ARG entry in the Comprehensive Antibiotic Resistance Database, we extracted the full list of drug classes and antibiotic names reported. Each unique combination adds one to the appropriate bar. Consequently, a single gene contig can contribute to several drug-class bars in panel A and several antibiotic bars in panel B.

Drug class analysis revealed diverse resistance profiles across different samples (Figure 3A). Container samples showed the most extensive resistance. Co-A exhibited resistance to seven drug classes, including fluoroquinolones, phenicols, diaminopyrimidines, and carbapenem antibiotics. Similarly, Co-C showed resistance to six drug classes, dominated by fluoroquinolones, sulfonamides, and phenicol antibiotics. In water samples, the resistance to aminoglycoside antibiotics, specifically streptomycin, was prevalent in Wo-A, Wi-A, and Wi-B (Figure 3B). Container samples displayed a higher diversity of resistance. Co-C demonstrated resistance to multiple sulfonamide variants alongside fluoroquinolones and phenicol antibiotics. In contrast, Co-A exhibited a narrower resistance spectrum dominated by trimethoprim and chloramphenicol. Fish samples displayed limited resistance, with Fi-2 showing resistance to trimethoprim and chloramphenicol.

**Figure 4:**
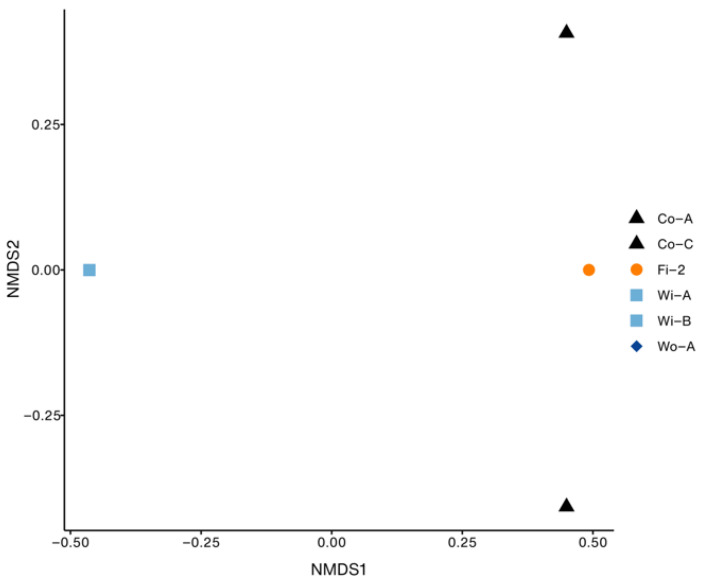
Non-metric multidimensional scaling (NMDS) plot based on Bray–Curtis distances showing the clustering of ARG gene families across water, container, and fish samples (stress = 0). Water samples cluster together, whereas containers and fish samples form a separate cluster.

The NMDS analysis based on Bray–Curtis distances revealed clustering patterns of ARG gene families across different samples (stress = 0.0; PERMANOVA, *p* = 0.1). ARGs detected in water samples (Wi-A, Wi-B, and Wo-A) clustered together, whereas container samples (Co-A and Co-C) and fish samples (Fi-2) formed a separate cluster (Figure 4).

## 4. Discussion

This study indicated the distinct shifts in the bacterial community composition along the Asian seabass supply chain, potentially influencing the transmission of ARGs. In water samples, *Synechococcus* was the dominant genus, but the detection of *Vibrio* species in the recirculating aquaculture system of Farm C suggested that intensive farming practices can facilitate pathogen proliferation. This observation is aligned with the previous report of increased *Vibrio* counts in aquaculture environments with high stocking densities [36].

A marked transition in the bacterial community was observed in transport containers, characterized by an increase in *Kocuria*, *Kytococcus*, *Acinetobacter*, *Shewanella*, and *Psychrobacter*, suggesting environmental contamination during handling [37,38,39]. Similar transitions were also observed in processing environments, where *Acinetobacter* increased from 3% in primary production to 34% in processing facilities [39]. The persistence of *Acinetobacter* and *Shewanella* in market samples indicated their ability to survive processing steps. Notably, *Acinetobacter* species are dominant in fish samples and exhibit resilience and antibiotic resistance [37,38]. For example, *A. johnsonnii* caused disease in the rainbow trout, *Oncorhynchus mykiss*, and exhibited resistance to antibiotics such as ampicillin [40], whereas *Acinetobacter baumannii* showed high abundance in fish and seafood with resistance to tetracycline, ampicillin, and gentamicin [41]. In addition, other bacteria, such as *Shewanella* and *Aeromonas,* were highly prevalent and have been known to induce pathogenicity in several fish species [42,43].

Although a relatively low prevalence of foodborne pathogens such as *Salmonella*, *Campylobacter*, *Listeria*, *Brucella*, *Yersinia*, and *S. aureus* was detected, their presence indicates a potential health risk that leads to foodborne illness if fish are inadequately cooked [44]. In addition, the high abundance of *E. coli* in some fish samples suggested fecal contamination at some point in the supply chain. *E. coli* contamination in fish is known to originate from water or contaminated ice [45,46]. The present results underline the importance of sanitary standards in food supply chain management.

Metagenomic analysis revealed distinct ARG distribution patterns across the sample types, suggesting different selective pressures and transmission mechanisms along the supply chain. A particular finding was the predominance and exclusivity of the *rpsL* gene, conferring resistance to streptomycin, in the water samples. This observation aligns with previous research indicating a high prevalence (95%) of *rpsL* in aquaculture environments [47]. The high prevalence, specifically in the water samples from Farms A and B, raises questions about its origin. While streptomycin has a long history of use in human medicine, particularly for tuberculosis treatment [48], and mutations in *rpsL* are known in *Mycobacterium tuberculosis* strains in Thailand [49], its widespread use in Thai aquaculture is not well documented. Several factors could contribute to the observed *rpsL* prevalence in the water. Firstly, historical use of streptomycin or other aminoglycosides in aquaculture or surrounding agriculture could have led to the selection and persistence of this gene in environmental bacteria residing in the water column or sediments. Secondly, the source water itself might carry this resistance signature from upstream contamination sources. The detection of *rpsL* in environmental water samples has been reported previously [50,51,52], and our study confirms its presence in Thai aquaculture water. It is important to note that while aminoglycosides are commonly used in aquaculture, our study did not detect other common aminoglycosides such as gentamicin and kanamycin.

In contrast to the water samples, transport containers emerged as ARG hotspots, harboring a broader and different array of ARGs. These included genes conferring resistance to multiple drug classes such as fluoroquinolones, phenicols, sulfonamides, diaminopyrimidines, and beta-lactams, including carbapenems (*bla*_OXA−436_ and *bla*_EAM-1_) and other variants (*bla*_OXA−504_, *bla*_OXA−661_, *bla*_OXA−912_, and *bla*_MOX-13_). This shift in ARG profile strongly suggests that post-harvest handling and transportation are critical points for ARG accumulation and potential transmission. The contamination sources are likely multifactorial, including residual water, ice, equipment, and cross-contamination during transportation [53]. Furthermore, the use of antibiotics in water during the transportation of live animals to maintain freshness or prevent mortality may exacerbate the selection and emergence of antibiotic-resistant bacteria [54]. The frequent detection of the *rsmA* gene, encoding a resistance-nodulation-cell division (RND) efflux pump known to confer resistance to diverse antibiotic classes [55], particularly in container sample Co-A and fish sample Fi-2, further underlines the high-risk nature of this stage. Although *rsmA* provides broad antibiotic resistance [47], its presence in container samples alongside specific fluoroquinolone and beta-lactam resistance genes indicates that these drug classes are the likely cause or reason for the resistance observed. The absence of aminoglycoside-specific resistance genes (such as *rpsL*) reinforces the idea that aminoglycosides were probably not the main driver behind the resistance in these samples. The distinct clustering of ARG profiles from water and those from containers and fish (Figure 4) reinforces the idea of different selective environments and contamination events occurring after fish are removed from the farm environment.

The potential spread of AMR through the food chain was particularly of concern due to the detection of carbapenem resistance genes (e.g., *bla*_EAM-1_ and *bla*_OXA-436_) in container samples. The presence of *bla*_OXA-48_-like genes, such as *bla*_OXA-436_, has been associated with treatment-resistant infections in both aquaculture and clinical settings [56]. The co-occurrence of these genes with other beta-lactamases, such as *bla*_MOX-13_, suggested the potential for multiple resistance mechanisms, which could further limit treatment options. Notably, the predominance of *bla*_OXA-48_-like genes in container samples rather than in fish or water underscored that post-harvest activities might be the primary stage for resistance acquisition. This observation aligned with findings in the pork production chain, where beta-lactamase genes were more prevalent on meat contact surfaces compared with end products [57].

A key finding of this study is the large difference between the many ARGs found in transport containers and the few ARGs detected in the fish samples handled within them. This difference likely results from several connected factors acting throughout the supply chain. First, the fish might have started with fewer ARGs from the farm, meaning less AMR to develop. This idea is supported by finding the main water-associated ARG (*rpsL*) only in water samples, not later in the fish. Second, the part of the fish we sampled is important. Our analysis focused on external tissues like gills and skin mucus, not the fish gut, which holds the largest amount of ARGs in fish [58,59]. Third, post-harvest handling and transport expose fish to container microbes. However, rapidly chilling the fish on ice likely slows down bacterial growth and might reduce the transfer of ARGs between bacteria on the fish surface [60,61]. At the same time, our study revealed that container surfaces are a suitable environment for bacteria carrying diverse ARGs to survive and accumulate, making the containers effective ARG reservoirs. The detection of the *rsmA* gene in fish and container samples, but not in water samples, and the clustering of these samples observed in NMDS indicated the contamination during handling. Together, these factors, including initial farm conditions, the specific fish parts sampled, post-harvest handling effects, the container environment favoring ARG persistence, inefficient transfer to fish, and analytical limits, provide a comprehensive explanation for why fish samples carried far fewer ARGs than the transport containers. In addition, the absence of other ARGs in other samples suggests that antibiotic usage in aquaculture was low compared with other meats [62].

## 5. Conclusions

The metagenomic analysis of ARGs in the Asian seabass supply chain indicated that post-harvest handling and transport were important control points for preventing antibiotic-resistant bacteria in the food system. ARGs and foodborne pathogens, including *E. coli* and *Salmonella*, were detected, suggesting potential public health risks. These findings emphasize the need for improved biosecurity measures, particularly during post-harvest handling. The utility of a One Health approach in tackling AMR in aquaculture, underscoring the interconnectedness of environmental, animal, and human health, should be implemented. Ongoing surveillance and targeted interventions, such as enhanced sanitation, cautious antibiotic use, and rapid detection of resistance, are essential to mitigate the spread of resistant pathogens in aquaculture.

## Figures and Tables

**Table 1:** Sampling locations, coordinates, and collection dates of the samples. Water samples represent water inside (Wi) and outside (Wo) fish cages, and container samples (Co) correspond to storage containers at the respective farms. Fish samples (Fi) were obtained from local markets.

Sample	Location	Coordinates	Date	Note
**Farm**				
**Wi-A, Wo-A**	Farm A	7.151493 N, 100.550689 E	18 June 2024	Cage, fish farm
**Wi-B, Wo-B**	Farm B	7.167891 N, 100.583121 E	21 June 2024	Cage, fish farm
**Wi-C, Wo-C**	Farm C	7.262975 N, 100.423502 E	25 June 2024	Recirculating aquaculture
**Container**				
**Co-A**	Farm A container	7.151493 N, 100.550689 E	18 June 2024	
**Co-B**	Farm B container	7.167891 N, 100.583121 E	21 June 2024	
**Co-C**	Farm C container	7.262975 N, 100.423502 E	25 June 2024	
**Market**				
**Fi-1**	Plaza market	7.012232 N, 100.466658 E	9 July 2024	
**Fi-2**	Plaza market	7.012232 N, 100.466658 E	9 July 2024	
**Fi-3**	Koh Mhee market	7.052951 N, 100.506005 E	10 July 2024	
**Fi-4**	Koh Mhee market	7.052951 N, 100.506005 E	10 July 2024	
**Fi-5**	Songkhla market	7.200978 N, 100.593816 E	11 July 2024	
**Fi-6**	Khokrai market	7.150168 N, 100.570592 E	11 July 2024	
**Fi-7**	Supermarket A	6.993322 N, 100.486395 E	11 July 2024	
**Fi-8**	Klongrian market	7.000037 N, 100.489579 E	17 July 2024	

## Data Availability

Raw reads were deposited on the NCBI SRA under the project number PRJNA1158344.

## Supplementary Materials

The following supporting information can be downloaded at: https://www.mdpi.com/article/10.3390/foods14101691/s1, Table S1: Total raw reads generated from sequencing, the number of reads that passed quality control, and the corresponding percentage. Table S2. Taxonomic classification of bacterial reads classified using Kraken2.
